# Electrochemical Sensor Capable of Enhancing Dopamine Sensitivity Based on Micron-Sized Metal–Organic Frameworks

**DOI:** 10.3390/bios15060348

**Published:** 2025-05-30

**Authors:** Ruhui Yan, Yuewu Zhao, Huaixiao Geng, Mengxia Yan, Jine Wang, Shuang Han

**Affiliations:** 1 School of Science, Shenyang University of Chemical Technology, Shenyang 110042, China; 2022092@stu.syuct.edu.cn (R.Y.); 2024139@stu.syuct.edu.cn (H.G.); 2 Shandong Provincial Engineering Laboratory of Novel Pharmaceutical Excipients, College of Pharmacy, Sustained and Controlled Release Preparations, Dezhou University, Dezhou 253023, China; mxyan@dzu.edu.cn

**Keywords:** metal–organic frameworks, graphene oxide, sheets, electrochemical, dopamine

## Abstract

Micron-sized, ultrathin metal–organic framework (MOF) sheet is a two-dimensional (2D) hybrid material with a large specific surface area, which can be used not only in the fields of energy and biomedicine, but also in electrode modification to improve the electrochemical detection effect. In this work, the 2D-structured Co-TCPP(Fe) MOF sheets were synthesized from porphyrin molecules and cobalt ions and then combined with reduced graphene oxide (rGO) and perfluorosulfonic acid polymer (Nafion) solution to construct Co-TCPP(Fe)/rGO/Nafion-modified electrodes capable of sensitively capturing dopamine (DA). The 2D ultrathin lamellar structure of this electrode-modified material is beneficial to the formation of π-π stacking effect with DA molecules, and the oxygen-containing groups carried on its surface can also form electrostatic attraction with the amino groups of DA molecules. Therefore, the Co-TCPP(Fe)/rGO/Nafion-modified electrode under the synergistic effect shows a specific adsorption effect on DA molecules, resulting in high anti-interference ability and a low detection limit of 0.014 µM in the concentration range of 0.1–100 µM. Furthermore, the Co-TCPP(Fe)/rGO/Nafion composite material composed of micron-sized, ultrathin lamellar structures also shows high reusability due to the stability of its coordination structure and can demonstrate good results when applied to the actual sample detection of human urine.

## 1. Introduction

Dopamine (DA) is one of the important neurotransmitters that control the central nervous system, and it mainly participates in basic physiological processes such as emotion, learning, and sleep through its characteristics as an extracellular chemical messenger [1]. Abnormal concentrations of DA in the human body can easily lead to the occurrence of various neurological diseases such as Alzheimer’s, Parkinson’s, Huntington’s, and schizophrenia [2,3]. According to the data of the Global Burden of Disease, Injuries, and Risk Factors Study (GBD) in 2021, about 3.4 billion people in the world were affected by nervous system diseases, which has increased by 18% in 31 years, resulting in the loss of about 443 million healthy years of life lost and becoming one of the major diseases of global burden [4]. Specifically, Alzheimer’s disease affects over 30 million people worldwide, Parkinson’s disease has approximately 10 million patients globally, Huntington’s disease accounts for 2.7 out of 100,000 people, and there are more than 21 million schizophrenia patients [5,6]. These diseases cause an economic burden of more than 600 billion dollars every year. As a catecholamine neurotransmitter containing a catechol structure, DA is prone to lose two electrons to form dopamine-o-quinone under electrochemical oxidation conditions, generating characteristic electrochemical signals [7]. Therefore, the dynamic changes of DA can be sensitively monitored by electrochemical methods. Compared with chromatography or spectroscopy, electrochemical detection only requires an extremely small amount of samples to achieve highly sensitive detection. These characteristics are more conducive to the monitoring of neurodegenerative diseases.

Materials that can identify DA are the basis of improving electrochemical detection performance, so it is indispensable to design and obtain highly sensitive recognition materials that can effectively modify electrodes. Porphyrin molecules are a class of compounds with planar and highly conjugated macromolecular heterocyclic structures, which can interact with the benzene ring of DA through π-π stacking [8]. Compared with solid particle materials, coordinating and combining porphyrin molecules with metal ions to construct metal–organic framework (MOF) material with a porous structure will have more advantages in exerting the efficacy of porphyrin molecules themselves. The MOF structure constructed by porphyrin molecules has a large specific surface area, high porosity, and regular topological structure, which makes it not only widely used in fields such as gas storage and release [9], catalysis [10,11], and cancer treatment [12,13,14], but allows it to achieve good results in the field of electrochemical applications [15,16,17]. In addition, metal ions can also enter the center of porphyrins to form metalloporphyrin, thus further enhancing the redox activity of the material in electrochemical detection [18]. Porphyrin can construct sensing materials by functionalizing alkane body molecules and combining with carbon nanomaterials. It can also be integrated into the device structure by using field effect transistors, modified electrodes, and other strategies for electrochemical sensing of ions and biomolecules [19]. The use of porphyrin-based MOF materials, especially two-dimensional, ultrathin MOF lamellar structure materials, is expected to enhance the π-π stacking effect with DA. In addition, MOF structural materials with carboxyl groups can also form electrostatic attraction with DA molecules with amino groups, which can further increase the enrichment effect of DA on the electrode surface. However, the relatively weak conductivity of porphyrin MOF limits its effective application in electrochemistry, and other related materials with higher electrical conductivity need to be added for assistance. The high electrical conductivity and significant specific surface area of graphene enable it to effectively enhance the performance of charge transfer [20,21]. Thus, combining graphene with a layered structure with MOF can enhance the interaction reaction with DA on the basis of high electrical conductivity.

**Scheme 1:**
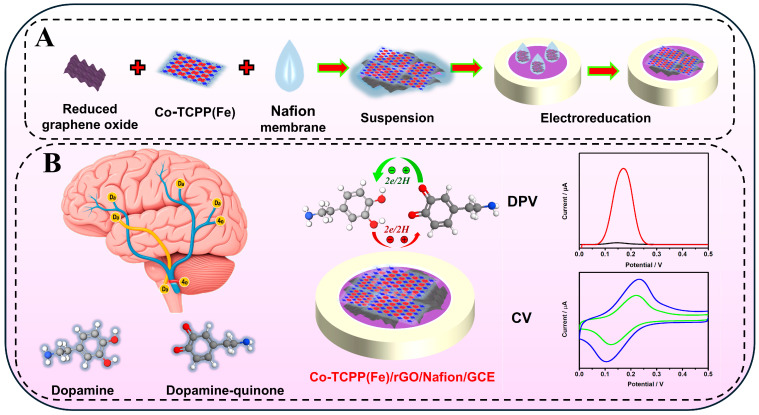
(**A**) Preparation process of a Co-TCPP(Fe)/rGO/Nafion-modified electrode and (**B**) the electrochemical detection of the DA application diagram.

Based on these considerations, as shown in Scheme 1, a two-dimensional structure of a MOF material was constructed by combining porphyrin with Co ions by a bottom-up reaction method, and then it was combined with graphene of a similar structure, thereby enhancing the enrichment effect of DA under the condition of increasing conductivity. Finally, the perfluorosulfonic acid polymer (Nafion) solution with selective permeability and electrode protection effect has been added to obtain the Co-TCPP(Fe)/rGO/Nafion-modified electrode. This electrode can act on DA molecules through the synergistic effect of electrostatic attraction and π-π stacking, presenting sensitive electrochemical detection signals within a wide concentration range and at low concentrations. Furthermore, the strong attraction effect of the electrode on DA indirectly enhances the anti-interference performance of the composite material against foreign molecules. On the other hand, due to the larger-sized layer structure of Co-TCPP(Fe) and rGO, the Co-TCPP(Fe)/rGO/Nafion/GCE electrode constructed by them also exhibits higher stability and reusability.

## 2. Experimental Section

### 2.1. Chemicals

Fe(III) meso-tetra(4-carboxyphenyl)porphine chloride [TCPP(Fe)] was obtained from Shanghai Tensus Biotech Co., Ltd. (Shanghai, China). Cobaltous nitrate hexahydrate [Co(NO_3_)_2_·6H_2_O], and dopamine were purchased from Aladdin Biochemical Co., Ltd. (Shanghai, China). The rGO solution was prepared by the modified Hummers method [22]. Uric acid and ascorbic acid were obtained from Shanghai Maclean Biochemical Co., Ltd. (Shanghai, China). The Nafion solution was purchased from Sigma-Aldrich Chemical Co., Ltd. (Shanghai, China). All other chemicals were analytical grade without further purification. The water used in the experiment is ultrapure water.

### 2.2. Synthesis of Co-TCPP(Fe) MOF Materials

Dissolve Co(NO_3_)_2_·6H_2_O and TCPP(Fe) in the *N*,*N*-dimethylformamide (DMF) solution to make their concentrations 10 mM and 5 mM, respectively. Subsequently, 15 mL of Co(NO_3_)_2_·6H_2_O solution, 15 mL of TCPP(Fe) solution, and 20 mL of DMF solution were added to a 100 mL round-bottomed flask and mixed evenly. The reaction was stirred at 100 °C for 4 h, then centrifuged and washed with ethanol to obtain the Co-TCPP(Fe) MOF products.

### 2.3. Synthesis of Co-TCPP(Fe)/rGO/Nafion Composite Materials

The 1.0 mg of Co-TCPP(Fe) was mixed with rGO solution (the concentration ratios are 2:1, 1:1, and 2:3, respectively), and then 100 μL of Nafion solution was added. After ultrasonic treatment of this mixed solution for 1 h, the Co-TCPP(Fe)/rGO/Nafion composite materials with different concentration ratios were obtained. Furthermore, by fixing the concentrations of Co-TCPP(Fe) and rGO solution, 50 μL and 70 μL of Nafion solution were added, respectively. After ultrasonic mixing, the Co-TCPP(Fe)/rGO/Nafion composite materials containing different amounts of Nafion were obtained.

### 2.4. Preparation of Co-TCPP(Fe)/rGO/Nafion-Modified Electrode

Polishing the glassy carbon electrode with Al_3_O_4_ powder, ultrasonic cleaning, and N_2_ drying to obtain the electrode with a clean surface. 1.0 mg/mL Co-TCPP(Fe) solution was evenly dispersed by ultrasound, and 5 μL was dripped on the surface of the electrode, which was naturally dried at room temperature to obtain a Co-TCPP(Fe)-modified electrode. The in situ electrochemical reduction of the material was carried out in PBS buffer (pH 7.0) deaerated with N_2_. The timing current method was used for scanning at a potential of −0.9 V for 100 s to obtain a stable curve. Other modified electrodes such as Co-TCPP(Fe)/rGO and Co-TCPP(Fe)/rGO/Nafion also utilize the same steps. The schematic outline for the preparation process of Co-TCPP(Fe)/rGO/Nafion-modified electrode is depicted in Scheme 1A.

### 2.5. Electrochemical Analysis

The electrochemical tests were conducted in the experiment using cyclic voltammetry (CV) and differential pulse voltammetry (DPV) methods. (i) Electrode property test: The three-electrode system was immersed in a 5.0 mM K_3_[Fe(CN)_6_]/K_4_[Fe(CN)_6_] solution containing 0.1 M KCl. The potential scanning range of CV was −0.2 to 0.6 V, and the scanning rate was 0.05 V/s. (ii) Sensor performance test: The three-electrode system was immersed in PBS solution with/without a certain concentration of DA. The potential scanning range of CV was −0.2 to 0.6 V, and the scanning rate was 0.05 V/s. The DPV determination was conducted by scanning within the potential range of 0–0.5 V, and the oxidation peak of DA was obtained. (iii) DPV parameters: Pulse amplitude is 0.05 V, pulse width is 0.05 s, the pulse period is 0.5 s, and the voltage increase is 0.005 V. All the electrolyte solutions in the experiment were deoxygenated with N_2_.

### 2.6. Actual Samples

The actual samples were selected from the urine of healthy young people, which did not contain endogenous DA (No history of neurological or metabolic abnormalities or any psychotropic drug use). After the samples were left to stand for 24 h, the supernatant was taken and diluted 100 times with PBS buffer solution (pH 7.0). DPV scanning analysis was performed on the DA urine samples using the standard addition method.

### 2.7. Characterization

The morphology and elemental composition of MOF were investigated by using the Hitachi HT7700 transmission electron microscope (TEM, the electron acceleration voltage is 100.0 kV). X-ray photoelectron spectroscopy (XPS) was obtained by the Thermo Scientific K-Alpha+ spectrometer. The ultraviolet-visible absorption spectrum was obtained by a 1280 ultraviolet-visible spectrophotometer (Shimadzu, Kyoto, Japan). FT-IR analysis was conducted using Nicolet Nexus 470 (Thermo Fisher Scientific, Waltham, MA, USA). Electrochemical tests (CV, DPV) were conducted at the CHI 660E electrochemical workstation (Shanghai Chenhua, Shanghai, China). Three-electrode system: The working electrode is a glassy carbon electrode (GCE, glassy carbon diameter is 3 mm), the counter electrode is a platinum sheet electrode, and the reference electrode is a saturated calomel electrode.

## 3. Results and Discussion

### 3.1. Characterization of the Synthesized MOF Materials

**Figure 1:**
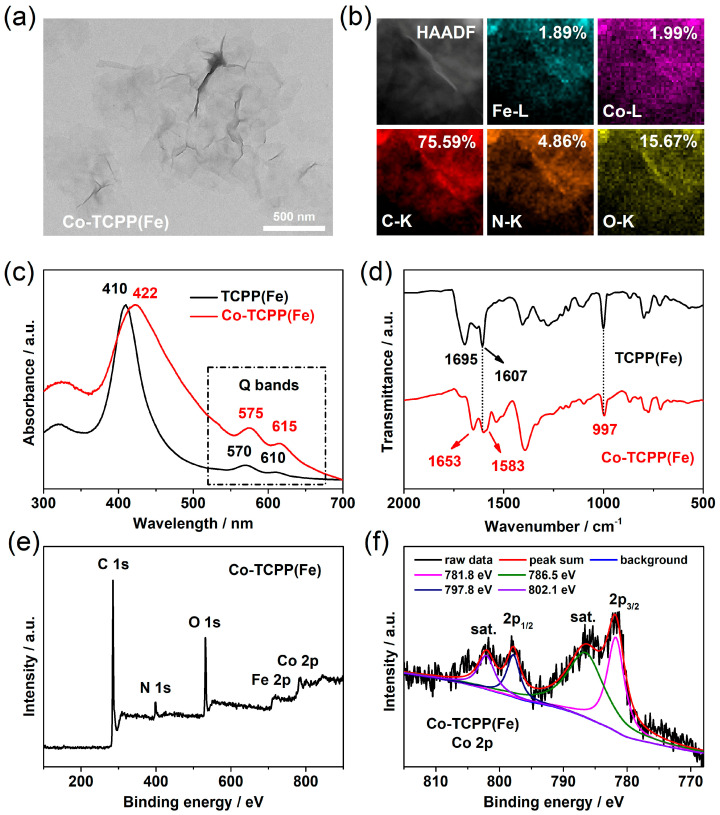
(**a**) TEM image, (**b**) elemental mapping, (**c**) UV-vis absorption, (**d**) FT-IR spectra, (**e**) full XPS, and (**f**) Co 2p spectra of the prepared Co-TCPP(Fe) MOF materials.

As shown in Figure 1a and Figure S1, the obtained Co-TCPP(Fe) material exhibits a two-dimensional, ultrathin lamellar morphology with micron-scale size, and the larger specific surface area generated by this special structure has great advantages for subsequent electrochemical detection applications. The corresponding element mapping in Figure 1b confirmed that C, N, O, Fe, and Co elements are homogeneously distributed in the Co-TCPP(Fe) materials, indicating that Co element and TCPP(Fe) undergo coordination reactions to form a periodic binding MOF structure. In addition, the organic ligands and the MOF products after the reaction were compared by spectroscopic analysis. As shown in Figure 1c, the UV-vis absorption of both TCPP(Fe) and Co-TCPP(Fe) showed two absorption peaks at the Q band position, which indicates that Fe is still in the central position of porphyrin before and after the coordination reaction. The Söret band absorption of Co-TCPP(Fe) was red shifted from 410 nm to 422 nm of the original TCPP(Fe) organic molecule, which further confirmed the formation of a MOF coordination structure. This spectral change due to the formation of the MOF structure is also reflected in FT-IR spectra. As shown in Figure 1d, with the formation of the MOF structure, the original C=O stretching vibration intensity of TCPP(Fe) at 1695 cm^−1^ was obviously reduced, and clear new peaks were formed at 1653 cm^−1^ and 1583 cm^−1^, which indicated that the carboxyl group of porphyrin had a coordination reaction with the Co element [23,24]. The remarkable N-Fe in-plane vibration at 997 cm^−1^ comes from the central position of the porphyrin ligand and has not changed, demonstrating that the coordination formation of the MOF structure does not affect the central structure of the porphyrin.

In order to further elucidate the microstructure of MOF materials, the obtained Co-TCPP(Fe) was analyzed by XPS. As shown in Figure 1e, the C, N, O, Fe, and Co elements obviously exist in Co-TCPP(Fe), which is consistent with the element mapping results. Among them, the binding energy peaks of Co 2p_3/2_ and Co 2p_1/2_ at 781.8 eV and 797.8 eV, accompanied by two satellite peaks at 786.5 eV and 802.1 eV (Figure 1f), confirm that the Co element is +2 valence state in the MOF structure, which is derived from the Co(II) ion reactants [25,26]. High-resolution C 1s also shows a significant COOR binding energy peak at 288.5 eV (Figure S2a), which is consistent with our previous results [13]. Because the porphyrin center of MOF materials is occupied by the Fe element, there is no -NH- binding energy peak in the N 1s of Co-TCPP(Fe), and only a binding energy peak of -C=N- is displayed at 398.5 eV (Figure S2b). These comprehensive characterization results indicate that Co ions can coordinate with the carboxyl groups of TCPP(Fe) to form MOF materials with an ultrathin layered structure, and the Fe element in porphyrin center cannot be replaced or substituted by Co ions in this reaction.

### 3.2. Electrochemical Performance of the MOF/rGO/Nafion Composites

**Figure 2:**
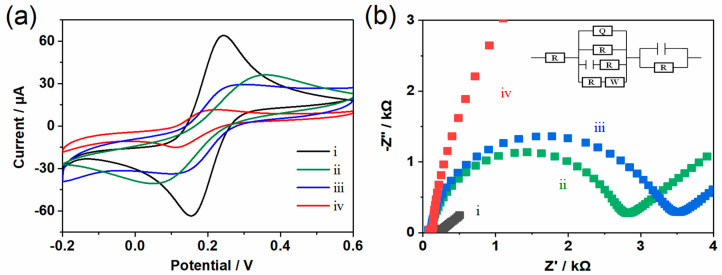
(**a**) CV plot and (**b**) EIS plot obtained in a 5.0 mM K_3_[Fe(CN)_6_]/K_4_[Fe(CN)_6_] solution containing 0.1 M KCl, where the scanning rate is 50 mV s^−1^. (i) GCE, (ii) Co-TCPP(Fe)/GCE, (iii) Co-TCPP(Fe)/rGO/GCE, and (iv) Co-TCPP(Fe)/rGO/Nafion/GCE.

The high electrical conductivity of materials is a prerequisite for electrochemical detection applications. In order to improve the electrochemical detection performance of MOF materials, the combination of Co-TCPP(Fe) and graphene may be able to obtain Co-TCPP(Fe)/rGO composites with high electrical conductivity. Here, the electrochemical performance of different modified electrodes was determined by the CV method. The redox probe was a 5 mM [Fe(CN)_6_]^3−/4−^ solution containing 0.1 M of KCl. Obviously, the probe has a pair of well-resolved redox peaks on the exposed GCE (Figure 2a, curve i), proving that the [Fe(CN)_6_]^3−/4−^ probe is a reversible process. After modifying GCE with MOF materials, its peak current intensity decreased significantly (Figure 2a, curve ii), indicating that the Co-TCPP(Fe) increased the impedance of the electrode. Contrary to expectations, the electrode modified by Co-TCPP(Fe)/rGO composite surprisingly generated a relatively low peak current (Figure 2a, curve iii). The possible reason is that although rGO has good electrical conductivity, the oxygen-containing groups it carries such as hydroxyl and carboxyl make it negatively charged, which will produce an electrostatic repulsive effect with [Fe(CN)_6_]^3−/4−^. The electrostatic repulsive force generated by adding molecules with negative charges was also confirmed after the addition of Nafion. As a cation exchange membrane, the negatively charged sulfonic acid groups of Nafion electrostatically repel the electrolyte solution, thereby causing the Co-TCPP(Fe)/rGO/Nafion-modified electrode to generate the relatively weakest current signal (Figure 2a, curve iv). Thus, it is speculated that the Co-TCPP(Fe)/rGO/Nafion-modified electrode might be able to generate a relatively high current detection capability for positively charged dopamine through electrostatic attraction.

The electrode performance and electrode interface phenomena were also studied by using the electrochemical impedance spectroscopy (EIS) method. A typical impedance spectrum (Nyquist diagram) is usually divided into two parts. The linear part of the EIS spectrum corresponds to the diffusion-limiting process, and the semi-circular part represents the electron transfer-limiting process. As shown in Figure 2b, with the addition of negatively charged rGO and Nafion, the resistance generated by the modified electrode gradually increases, and the result is consistent with that of CV. In addition, the proportion of MOF, rGO, and Nafion will also affect the performance of the composite material. As shown in Figure S3, when the concentration ratio of Co-TCPP(Fe) to rGO is 2:1, the Co-TCPP(Fe)/rGO/Nafion-modified electrode can produce a better current response to DA. Other proportions either have a smaller current response or are prone to detachment. Subsequently, the influence of Nafion dosage was compared and analyzed. Finally, a mixed solution with the concentration ratio of Co-TCPP(Fe) to rGO of 2:1 and the addition amount of 100 μL of Nafion was selected to construct the Co-TCPP(Fe)/rGO/Nafion composite material.

### 3.3. Detection DA of the MOF/rGO/Nafion Composites

**Figure 3:**
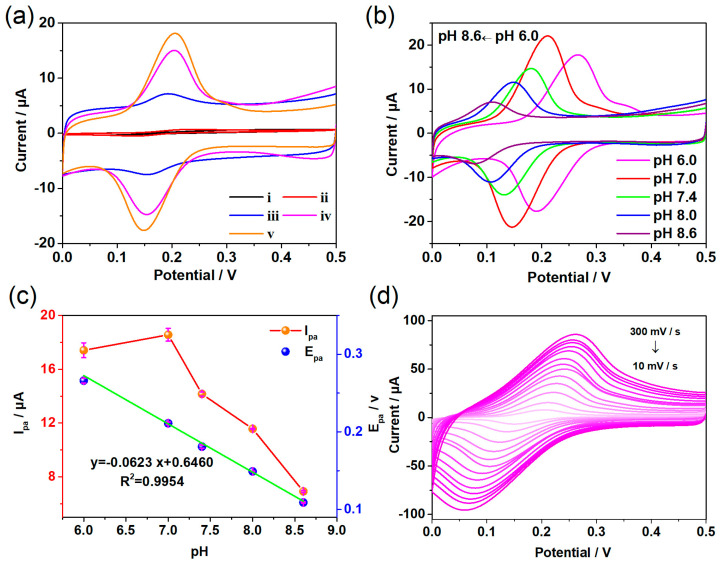
CV plots obtained in a 0.1 M PBS containing 50 µM DA, where the scanning rate is 50 mV s^−1^. (**a**) The different electrodes: (i) GCE, (ii) Co-TCPP(Fe)/GCE, (iii) rGO/GCE, (iv) Co-TCPP(Fe)/rGO/GCE, and (v) Co-TCPP(Fe)/rGO/Nafion/GCE. (**b**) CV results of the Co-TCPP(Fe)/rGO/Nafion/GCE at different pH, and (**c**) the changes of anode peak current I_pa_ and anode peak potential E_pa_. (**d**) CV results of the Co-TCPP(Fe)/rGO/Nafion/GCE at different scan rates.

To verify the electrochemical performance of the constructed composites, the electrochemical behavior of DA on several modified electrodes was investigated. As shown in the cyclic voltammetric (CV) curves of Figure 3a, the same amount (50 µM) of DA shows obvious differences in current intensity at different modified electrodes. The bare glass carbon electrode (GCE, curve i) has a very low current intensity and a pair of reversible redox peaks appeared in its CV curve. After being modified with Co-TCPP(Fe) (curve ii), the redox current was enhanced compared with that of bare GCE, which is attributed to the interaction between MOF and DA. Based on the weak conductivity of Co-TCPP(Fe) material, the electron transfer on the electrode surface is significantly limited; thus, the CV results only display a small current enhancement effect. In contrast, the rGO with strong conductivity can respond to DA obviously (curve iii), which may come from two aspects. First, the excellent electron conductivity of rGO accelerated the charge transfer between the electrode and the solution. Second, the unique two-dimensional, large surface area structure of graphene and its π-π interaction with DA molecules can effectively capture DA molecules in solution, thereby improving the detection signals. After combining MOF with rGO, the peak current of DA is further enhanced (curve iv), which is not only due to the role of rGO in electron transfer and molecular enrichment, but also because of the porous structure, high specific surface area, and specific adsorption ability of Co-TCPP(Fe) materials. The electrocatalytic performance and molecular recognition ability of the electrode surface has been optimized through the synergistic effect of MOF and rGO. The addition of perfluorosulfonic acid polymer (Nafion), which can hinder anions, further enhances the current of the composites (curve v). Compared with the bare GCE, the redox current intensity of DA in rGO/GCE, Co-TCPP(Fe)/rGO/GCE, and Co-TCPP(Fe)/rGO/Nafion/GCE increased by 11.1 times, 23.3 times, and 28.2 times, respectively. In addition, the results of DPV (Figure S4a) are consistent with those of the CV curves, which also confirms that Co-TCPP(Fe)/rGO/Nafion composites have stronger electrocatalytic oxidation capacity. Therefore, the special structure based on two-dimensional MOF, combined with rGO with excellent electrical conductivity, enables the Co-TCPP(Fe)/rGO/Nafion modified electrode to have good electrocatalytic behavior towards the DA.

The pH of solution may affect the electrocatalytic performance of Co-TCPP(Fe)/rGO/Nafion composite; thus, the electrocatalytic effect of this material for DA in the range of pH 6.0–8.6 was analyzed. As shown in Figure 3b–c, the peak current of redox reached its maximum value at pH 7.0, indicating that the Co-TCPP(Fe)/rGO/Nafion-modified electrode had the highest electrocatalytic activity for DA under neutral conditions. Both acidic and alkaline conditions will reduce the electrochemical activity of the Co-TCPP(Fe)/rGO/Nafion composites, especially with the increase in alkalinity. The reason for the sharp decline in electrochemical activity may be that the alkaline solution reduces the positive charge of DA, which hinders the electrostatic attraction with the composite material. However, the redox peak potential of DA showed an obvious overall negative shift trend in the range of pH 6.0–8.6, where the linear regression equation of oxidation peak potential is *y* = 0.646 − 0.0623*x* with the correlation coefficient of 0.995. The slope value (−62.3 mV) is close to the Nernstian theoretical value of −59 mV, indicating that the number of electrons and protons involved in the oxidation of DA on the surface of Co-TCPP(Fe)/rGO/Nafion/GCE is equal. Because the change in pH makes the concentration of protons different, the enhancement of alkaline conditions makes it easier to deprotonate. Thus, the redox peak potential tends to negative shift with the increase in pH value. Furthermore, the CV results generated by Co-TCPP(Fe)/rGO/Nafion/GCE electrode to 50 µM of DA at various scan rates were investigated. As shown in Figure 3d, the peak current intensity of redox not only increases with the enhance of scanning rate, but it is also proportional to the square root of scan rates from 10 to 300 mV s^−1^, suggesting that the electrochemical behavior of the Co-TCPP(Fe)/rGO/Nafion interface belongs to a reversible and diffusion-controlled reaction.

### 3.4. Detection Sensitivity of the MOF/rGO/Nafion Composites

**Figure 4:**
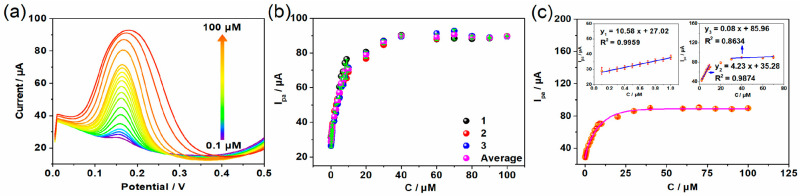
Co-TCPP(Fe)/rGO/Nafion/GCE at different concentrations of DA. (**a**) DPV curves, (**b**) data from three determinations and mean data with error bars, and (**c**) concentration linearity.

Since the Co-TCPP(Fe)/rGO/Nafion-modified electrode can produce better electrochemical behavior, the electrochemical detection range and detection limit of the composite material for DA were analyzed. As shown in Figure 4a, the oxidation peak current of DA in the concentration range of 0.1–100 µM on the Co-TCPP(Fe)/rGO/Nafion-modified electrode was determined by the DPV method, and its intensity showed a regular increase with the enhancement of DA concentration. Moreover, the results of different electrodes constructed by the same method all have the same growth trend (Figure 4b), indicating that the Co-TCPP(Fe)/rGO/Nafion material has extremely high stability and repeatability in electrochemical detection. Based on the results of several electrochemical tests, the linear regression equations were *y* = 27.02 + 10.58*x*, *y* = 35.28 + 4.23*x*, and *y* = 85.96 + 0.08*x* in the concentration ranges of 0.1–1.0 µM, 2.0–10 µM, and 30–100 µM, respectively (Figure 4c). Furthermore, the detection limit obtained based on a signal-to-noise ratio of 3 is 0.014 µM, which is superior to most other reported composite materials (Table 1). The reason may be that the ultrathin MOF material with a two-dimensional structure constructed by porphyrin not only has a large specific surface area but also a macrocyclic conjugated structure of its organic molecules, thus providing a rich electron cloud that can adsorb DA with an aromatic ring structure through π-π stacking interaction. Moreover, the carboxyl groups of the MOF material and the oxygen-containing groups carried by rGO can also form electrostatic attraction interactions with the amino groups of DA, further promoting the enrichment of DA on the electrode surface. Of course, the excellent electron transfer function of rGO in this composite material is also an indispensable factor.

### 3.5. Stability and Selectivity of the MOF/rGO/Nafion Composites

**Figure 5:**
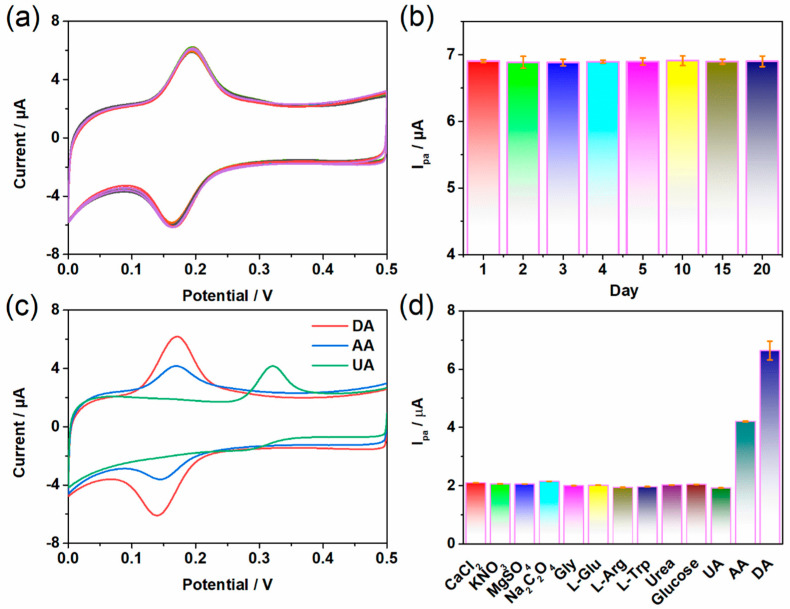
(**a**) CV curves of Co-TCPP(Fe)/rGO/Nafion/GCE after 30 cycles at 5 µM DA. (**b**) Peak current response obtained by the Co-TCPP(Fe)/rGO/Nafion/GCE sensor constructed by the same method under multiple tests at 5 µM DA. CV diagrams of 5 µM DA in the presence of 250 µM of other interfering substances (**c**) UA and AA, and (**d**) CaCl_2_, KNO_3_, MgSO_4_, Na_2_C_2_O_4_, Gly, L-Glu, L-Arg, L-Trp, Urea, and Glucose.

The stability, reproducibility, and anti-interference ability of the material are also important indicators for electrode evaluation. As shown in Figure 5a, the CV curves of 5 µM DA after 30 cycles under a Co-TCPP(Fe)/rGO/Nafion-modified electrode did not show significant attenuation, indicating that the composite material has excellent current stability. Moreover, the electrode can be used multiple times over a relatively long period of time (Figure 5b), and its current intensity does not decrease significantly, suggesting that the Co-TCPP(Fe)/rGO/Nafion composite has good reusability. To investigate the selective detection ability of Co-TCPP(Fe)/rGO/Nafion-modified electrode for DA, the experiment focused on studying the effects of uric acid (UA) and ascorbic acid (AA), which have a greater interference with the determination of DA. As shown in Figure 5c, the oxidation peak current intensity of Co-TCPP(Fe)/rGO/Nafion-modified electrode at a concentration of 5 µM DA is approximately 6.19 µA, while that at a concentration of 250 µM AA is only about 4.17 µA. Under a huge concentration difference of 50 times, the oxidation peak current intensity of AA is still much lower than that of DA, which proves that AA does not have an obvious impact on the detection of DA. In addition, the oxidation peak potential of UA is significantly different from the result of DA, and its smaller current intensity cannot cause interference either. Because actual samples often contain multiple ions or chemical substances, the interference of other foreign substances has also been analyzed (Figure 5d). In the presence of 50 times the external interfering substances such as CaCl_2_, KNO_3_, MgSO_4_, Na_2_C_2_O_4_, Gly, L-Glu, L-Arg, L-Trp, Urea, and Glucose, no obvious interfering peak current was observed on the Co-TCPP(Fe)/rGO/Nafion-modified electrode. These results indicate that the electrode modified with Co-TCPP(Fe)/rGO/Nafion can not only produce a more sensitive electrochemical detection effect on DA, but it also has a strong anti-interference performance.

### 3.6. Detection of Real Samples of Human Urine

To evaluate the performance and applicability of the constructed Co-TCPP(Fe)/rGO/Nafion composite material, the modified electrode was used to analyze real samples of human urine. Human urine was diluted 100 times, and the recovery rate was analyzed by adding the standard amount of DA. As can be seen in Table 2, the recovery rate of the labeled samples is within the range of 94.58–102.71%, indicating that the Co-TCPP(Fe)/rGO/Nafion-modified electrode also has good accuracy when applied to the detection of real samples. These results confirmed that the Co-TCPP(Fe)/rGO/Nafion composite material not only has high stability and selectivity in the electrochemical detection of DA, but also its high anti-interference ability makes the modified electrode have broad prospects in the application of actual samples.

## 4. Conclusions

In summary, an electrochemical sensor that can be effectively applied to DA was constructed by using two-dimensional structured MOF and rGO. The stability, reproducibility, and anti-interference performance of a Co-TCPP(Fe)/rGO/Nafion-modified electrode enable it to sensitively identify DA in the concentration range of 0.1–100 µM and have a lower detection limit of 0.014 µM. The large specific surface area of two-dimensional MOF structural materials can enhance the π-π stacking effect of DA on the surface. The negative charge property of oxygen-containing groups can form electrostatic attraction with DA. Combined with rGO, which has excellent electron conduction performance, the obtained Co-TCPP(Fe)/rGO/Nafion/GCE thus has a high sensitivity to the DA enriched on its surface. In addition, this sensor can also be effectively applied to the actual sample detection of human urine and has a good effect. Therefore, combining two-dimensional structured MOF materials with rGO may have promising application prospects not only in electrochemical detection but also in gas sensing, electronic devices, and life sciences. There is still much space for exploration in the application of MOF in the field of neurotransmitters detection. Integrating MOF materials into portable electrochemical detection devices to achieve real-time monitoring or medical diagnosis may be a future direction. Introducing multifunctional ligands to construct composite probes capable of simultaneously detecting multiple metabolites may be another research outlet in the future.

## Figures and Tables

**Table 1:** Comparison of different modified electrodes for the determination of DA.

Electrode	LDR (μM)	LOD (μM)	Samples	Refs
GO-UiO-66-RT	2~200	2.1	0.5 M KCl	[27]
SO_3_-MOF-808	50~500	2.3	0.5 M KCl	[28]
Ce-MOF-808	20~200	1.3	---	[29]
AuPd/UiO-66-NH_2_/GN	0.9~300	0.21	Serum	[30]
CoNi-MOF@ERGO	0.1~400	0.086	Serum	[31]
ERGO-MnTMPyP	2.23~10.76	0.29	Serum	[32]
GO-Cu NPs-Fe-MOF/CPE	0.4~495.2	0.1	Serum, urine	[33]
ERGO/polyCoTAPc	2.0~100	0.095	urine	[34]
CoMOF-GO/1-M,3-BB	0.01~300	0.04	urine	[35]
TAPP@GO	0.1~300	0.038	urine	[36]
CoTCPP(Fe)/rGO/Nafion	0.1~100	0.014	urine	this work

**Table 2:** The results of DA detection in human urine samples (*n* = 3).

Sample	Added (μM)	Found (μM)	Recovery (%)	RSD (%)
1	3.000	2.84	94.58	2.19
2	4.000	3.95	98.87	0.66
3	5.000	5.10	102.71	0.82

## Data Availability

Data collected and/or analyzed during this study can be obtained from the corresponding author upon reasonable request.

## Supplementary Materials

The following supporting information can be downloaded at: https://www.mdpi.com/article/10.3390/bios15060348/s1, Figure S1: The large-scale TEM image of the Co-TCPP(Fe) materials.; Figure S2: (a) C1s spectra, and (b) N 1s spectra of the Co-TCPP(Fe) materials. Figure S3: (a) CV curves of Co-TCPP(Fe) with different ratios of Co-TCPP(Fe) to rGO. (b) CV curves of Co-TCPP(Fe)/rGO/Nafion/GCE in 50 μM DA with different Nafion additions (N50: Nafion 50 µL; N70: Nafion 70 µL; others are 100 µL Nafion additions). (c) Histograms of anode peak current under different conditions. Figure S4: (a) DPV plots of different electrodes measured with 0.1 M PBS under conditions containing 50 µM DA. (b) Under the condition of 50 μM DA, the linear relationship between the CV anode peak current I_pa_ of Co-TCPP(Fe)/rGO/Nafion/GCE at different scanning rates and the square root of the scanning rate.
