# Supplementary material for: Electrochemical Sensor Capable of Enhancing Dopamine Sensitivity Based on Micron-Sized Metal–Organic Frameworks
Source: Biosensors (Basel). 2025 May 30;15(6):348. doi: 10.3390/bios15060348 (PMC12190527; doi:10.3390/bios15060348)
Supplement: Supplementary file 1 [file biosensors-15-00348-s001.zip › biosensors-3644222-supplementary.pdf]

Supporting Information

Electrochemical Sensor Capable of Enhancing Dopamine Sensitivity Based on Micron-Sized Metal–Organic Frameworks

Ruhui Yan [1,2], Yuewu Zhao [2,*], Huaixiao Geng [1,2], Mengxia Yan [2], Jine Wang [2,*] and Shuang Han [1,*]

[1] School of Science, Shenyang University of Chemical Technology, Shenyang 110042, China; 2022092@stu.syuct.edu.cn (R.Y.); 2024139@stu.syuct.edu.cn (H.G.)

[2] Shandong Provincial Engineering Laboratory of Novel Pharmaceutical Excipients, College of Pharmacy, Sustained and Controlled Release Preparations, Dezhou University, Dezhou 253023, China; mxyan@dzu.edu.cn

* Correspondence: ywzhao2024@dzu.edu.cn (Y.Z.); wangje2024@dzu.edu.cn (J.W.); unihanshuang@syuct.edu.cn (S.H.)

Figure captions

Figure S1

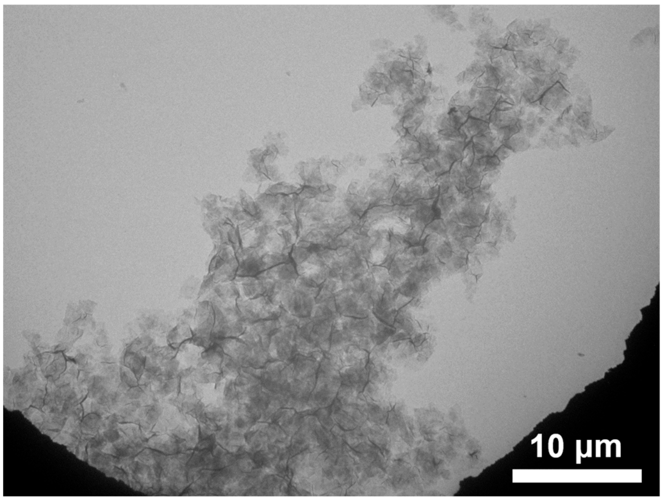

Figure S1. The large-scale TEM image of the Co-TCPP(Fe) materials.

Figure S2

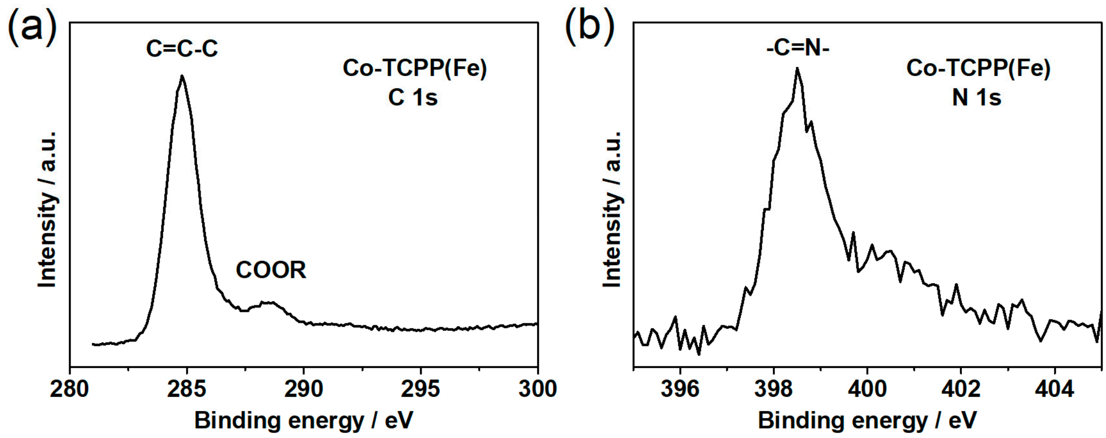

Figure S2. (a) C1s spectra, and (b) N 1s spectra of the Co-TCPP(Fe) materials.

Figure S3

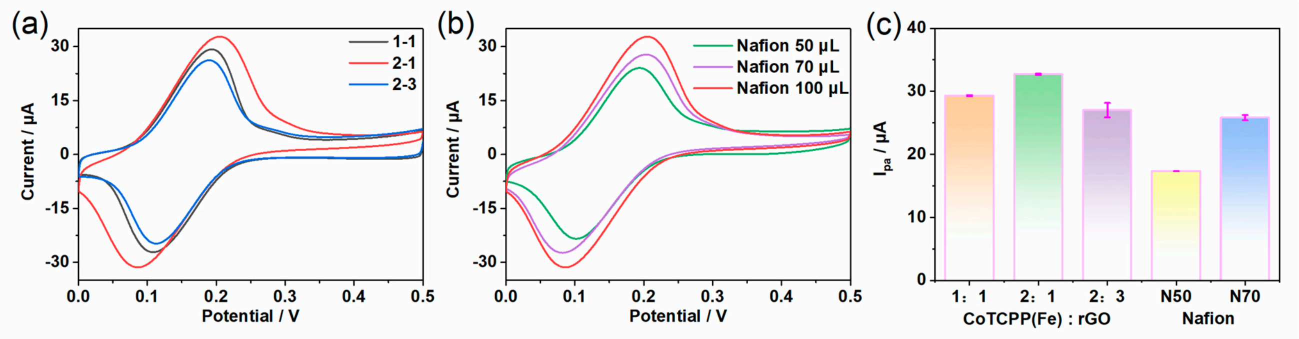

Figure S3. (a) CV curves of Co-TCPP(Fe) with different ratios of Co-TCPP(Fe) to rGO. (b) CV curves of Co-TCPP(Fe)/rGO/Nafion/GCE in 50 μM DA with different Nafion additions (N50: Nafion 50 μL; N70: Nafion 70 μL; others are 100 μL Nafion additions). (c) Histograms of anode peak current under different conditions.

Figure S4

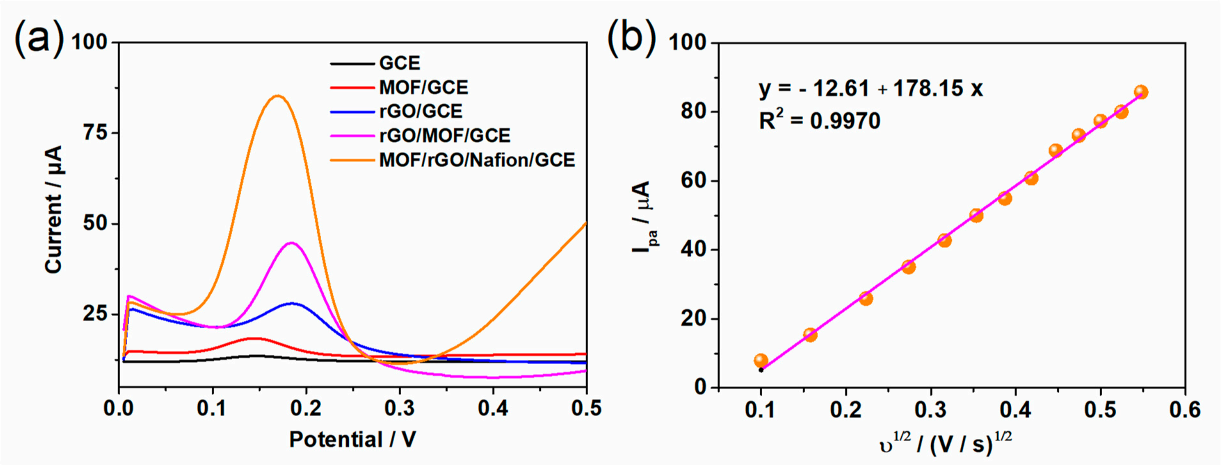

Figure S4. (a) DPV plots of different electrodes measured with 0.1 M PBS under conditions containing 50 μM DA. (b) Under the condition of 50 μM DA, the linear relationship between the CV anode peak current I_{pa} of Co-TCPP(Fe)/rGO/Nafion/GCE at different scanning rates and the square root of the scanning rate.
